# Results of bracing adolescent idiopathic scoliosis in the context of clinical practice and the Scoliosis Research Society’s criteria: 5-year observational study from a German orthopaedic university hospital

**DOI:** 10.1186/s40001-024-02112-y

**Published:** 2024-10-29

**Authors:** Heide Delbrück, Isabel Karl, Frank Hildebrand, Miriam K. Hertwig, Miguel Pishnamaz

**Affiliations:** Department of Orthopaedics, Trauma and Reconstructive Surgery, University Hospital RWTH Aachen, Pauwelsstrasse 30, 52074 Aachen, Germany

**Keywords:** Adolescent idiopathic scoliosis, Brace, AIS, SRS

## Abstract

**Background:**

Brace therapy’s influence on adolescent idiopathic scoliosis’s (AIS) natural course is inconclusive.

**Methods:**

Brace-treated AIS patients from 2016 to 2020 were examined regarding four endpoints at brace weaning: surgery need, curve progress ≥ 6° and > 45°, and curve improvement ≥ 6°. Prediction variables’ influence was computed for the all-patients group and three subgroups (Subgroup 1: fulfilling the Scoliosis Research Society’s [SRS] criteria, Subgroup 2: initial Cobb angle < 25°, Subgroup 3: initial Cobb angle > 40°). According to the data characteristics, parametric and non-parametric tests and binary logistic regression were performed.

**Results:**

The research included 69 patients. Overall, after brace weaning surgery was recommended for 20.3% of them, curve progression was ≥ 6° in 23.2%, the Cobb angle was beyond 45° in 11.6%, and the Cobb angle improved by ≥ 6° in 20.3%. Patients needing surgery had a significantly higher initial Cobb angle (38.8° ± 9.8° vs 27.8° ± 7.6°, *p* < 0.001), lower Risser stages (*p* = 0.010), and higher Nash and Moe degrees (*p* = 0.030). Patients with curve progress ≥ 6° were younger at first curve notification (12.4 ± 1.5 vs 13.7 ± 1.7 years, *p* = 0.011) and older at menarche (13.4 ± 1.1 vs 12.6 ± 1.2 years, *p* = 0.037). Furthermore, 21.6% of all Risser 3 and 4 patients had still curve progress ≥ 6°. In-brace correction was significantly higher in patients with curve improvement ≥ 6° vs < 6° (54.0% ± 31.2% vs 31.9% ± 30.7%; *p* = 0.019). Nine patients fulfilled the SRS criteria, 22 had initial Cobb angles < 25°, and 11 had > 40°. Real brace wear (RBW) in all groups had no significant effect on outcome. Two significant subgroup differences were found: Surgery recommendation and curve progression beyond 45° were significantly more frequent in the initial Cobb angle > 40° subgroup.

**Conclusions:**

Brace effectiveness can be assumed because curve improvement was achieved in nearly one-fifth with sufficient in-brace correction. However, no significant influence of RBW on the outcome endpoints was demonstrated. To clarify conflicting results, big data management, including untreated patients, must be employed to further research AIS’s multifactorial influenced aetiology and course. Meanwhile, it is worth starting bracing in AIS in practice also beyond the SRS’s criteria.

**Supplementary Information:**

The online version contains supplementary material available at 10.1186/s40001-024-02112-y.

## Introduction

Based on a South Korean nationwide database, the incidence of adolescent idiopathic scoliosis (AIS) in patients aged 10–14 years is 0.82%, and 1.14% of those affected underwent surgery within 5 years after initial diagnosis [1]. In their review based on studies from Europe, Singapore, Brazil and South Korea Konieczny et al. described an overall AIS prevalence of 0.47–5.2% [2]. In a meta-analysis, the pooled prevalence of spinal curves with a Cobb angle ≥ 10° was 1.34% (34 studies) and 0.2% with Cobb angle ≥ 20° (28 studies) [3].

In 117 untreated AIS patients after a 50-year follow-up, the Iowa natural history studies of AIS reported that 22% of untreated patients with AIS vs 15% of the age- and sex-matched control group without AIS (62 patients) complained of shortness of breath. An increased risk of shortness of breath was associated with a Cobb angle > 80° in combination with thoracic apex. Furthermore, 61% of untreated patients with AIS vs 35% of the matched control group without AIS reported chronic back pain. Major curves < 30° at skeletal maturity tended not to progress. Described factors determining curve progression were curve location and magnitude and skeletal maturity [4, 5]. Curves with a Cobb angle > 50° at skeletal maturity tend to progress further throughout adulthood, making this value a threshold for surgery recommendation [5–7]. This is why all efforts are aimed at keeping the curves below 50° until skeletal maturity is complete.

Today, external bracing and physiotherapy represent the major pillars of nonsurgical scoliosis treatment. In the multicentre study ‘Bracing in Adolescent Idiopathic Scoliosis Trial (BRAIST)’ (242 patients included), successful treatment was achieved in 72% of patients by bracing compared to 48% without bracing [8]. However, a Cochrane review to the topic ‘Braces for Idiopathic Scoliosis in Adolescents’ could only include seven studies (662 participants) for qualitative synthesis, concluding the evidence regarding brace treatment for AIS as low to very low quality. Until now, there are two randomized controlled clinical trials (RCTs) [8, 9] and two prospective cohort studies [10, 11] that compare bracing and observation and demonstrate that bracing is superior to observation. Thus, evidence of brace efficiency in AIS appears to be based on only a few studies with reasonable quality. However, conducting RCTs that compare bracing and observation is difficult, not least because of the parents who do not want to withhold any therapy from their children that could work.

Taking this fact into account, the International Society on Scoliosis Orthopaedic and Rehabilitation Treatment (SOSORT) and the SRS developed recommendations for research studies on conservative treatment of AIS. With clearly delineated inclusion criteria, methodologies, and outcome measures, better and easier future meta-analysis or comparative studies should be possible [12].

The aim of our retrospective observational study is to add treatment results of braced patients to synthesise studies considering the SRS and SOSORT guidelines. There is still no clarity in the literature on the predictors for progression of scoliosis, and the results are not coherent among studies [13]. Presenting detailed data of different patient cohorts could be helpful to analyse the influence of multiple variables and approaches [13]. Furthermore, the collected data should be made usable for the regularly necessary educational talks about brace success rates with parents and patients in our department.

## Methods

The present study was performed according to the Strengthening the Reporting of Observational Studies in Epidemiology guidelines (STROBE) [14].

### Patients

For this retrospective study, the medical records of all patients at RWTH Aachen University Hospital with the Diagnosis Related Groups (DRG) principal diagnosis of scoliosis were reviewed in the 5-year period from 2016 to 2020. Patients with AIS and brace therapy were selected.

### Brace

Patients were treated with Chêneau-Brace. The braces were constructed by a long-time experienced Certified Prosthetists Orthotist (CPO) who has already built more than 1000 braces. He completed the 3D Rigo-Chêneau-Brace practical seminar at the Bundesfachschule für Orthopädie-Technik in Dortmund/Germany, Germany's central college for Prosthetics & Orthotics. This seminar was led by Manuel Rigo [15]. SOSORT recommendations for team role in bracing have been established standards in our daily routine for years [7]. All patients received concurrent physiotherapy.

### Evaluated parameters

According to the efforts to optimise corset studies [16], the following parameters were evaluated: age at brace initiation (≙ age in first padded brace) and brace termination, Risser sign for skeletal maturity at brace initiation (according to ‘Risser + ’staging, [12]), curve size (Cobb angle of the largest curve) at initial diagnosis and in brace, curve magnitude grouping, gender, curve pattern, curve rotation (Nash and Moe), menarche status, and RBW. The Cobb angle at the time of the outcome measurements was measured in a brace-free X-ray at skeletal maturity after the brace was fully weaned. For describing curve patterns we used Ponseti et al.’s classification: lumbar, thoracolumbar, combined thoracic/lumbar, thoracic, and cervicothoracic [7, 17]. Measurements were done in accordance to the Radiographic Measurement Manual of the Spinal Deformity Study Group [18].

All patients were prescribed a full-time brace. RBW was evaluated based on the reports by the patients in the patient files and classified into four groups: 16–23 h, 8–16 h, < 8 h, and brace refused. The ‘brace refused’ group includes patients who were prescribed a brace, for whom it was made, and who also underwent the check-ups with the brace but did not wear it daily.

### Subgroups

The evaluation was conducted for the group that included all patients, with three subgroups classified from it:

- Subgroup 1: fulfilling the SRS criteria: above 10 years of age, Risser 0–2, curves 25–40° Cobb angle [12, 16]
- Subgroup 2: initial Cobb angle < 25°
- Subgroup 3: initial Cobb angle > 40°

### Endpoints

For assessment of brace effectiveness, the percentage of patients who have 5° or less curve progression and the percentage of patients who have 6° or more progression at skeletal maturity were determined. Furthermore, the percentage of patients with recommended/undergone surgery before and after skeletal maturity and patients who progress beyond a Cobb angle of 45° were documented. Curve improvement ≥ 6° was also evaluated. These four endpoints were calculated and considered independently of each other (i.e., there may also be, for example, patients in the ‘surgery recommended’ group who had no progression ≥ 6°). The surgery was recommended in the case of a Cobb angle of 40–50°, also considering the existing rotation and body asymmetry.

### Statistics

Statistical analysis was performed with IBM SPSS Statistics, Version 29.0.0.0. For metric variables, the Shapiro–Wilk test was used for the analysis of normal distribution. With the Levene test, the homogeneity of variance was checked. A *T* test was then performed to analyse the differences in mean in normally distributed variables. The Mann–Whitney *U* test was conducted for ordinal variables and in place of *T* test when the Shapiro–Wilk’s test was significant. A chi-squared test was used in the analysis of contingency tables. Binary logistic regression was performed for computing odds ratio (OR). The calculations for subgroup comparison were carried out with Fisher’s exact probability calculator of MedCalc [19].

## Results

### Patient selection

**Fig. 1:**
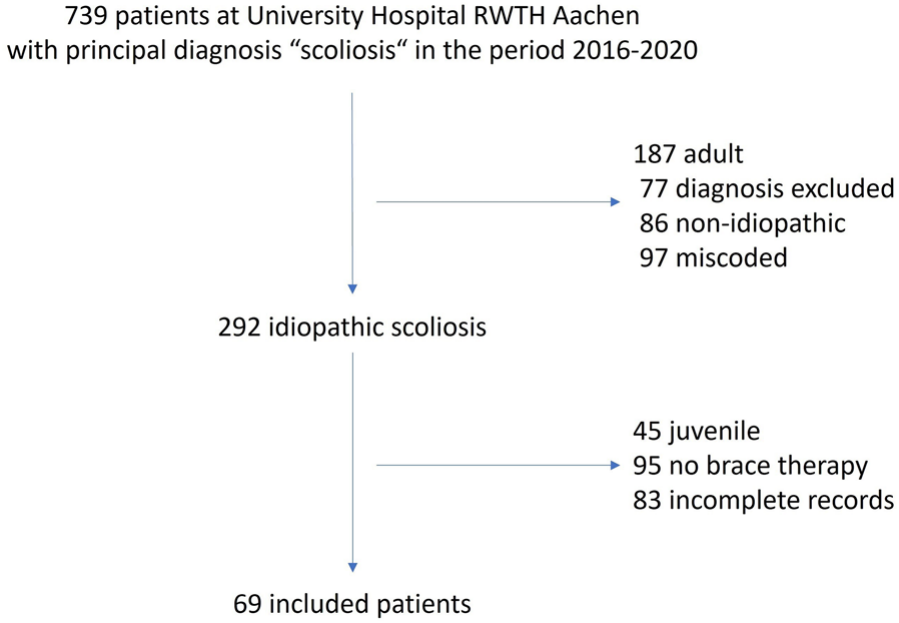
Patient selection

Overall, 739 patients with DRG principal diagnosis of scoliosis were treated from 2016 to 2020. After excluding patients with non-idiopathic and adult scoliosis and incorrect coding, 292 patients with idiopathic scoliosis remained. Finally, 69 patients were included in the research after excluding patients with juvenile scoliosis, patients without brace therapy, and cases with incomplete records (Fig. 1).

### Patient characteristics

**Fig. 2:**
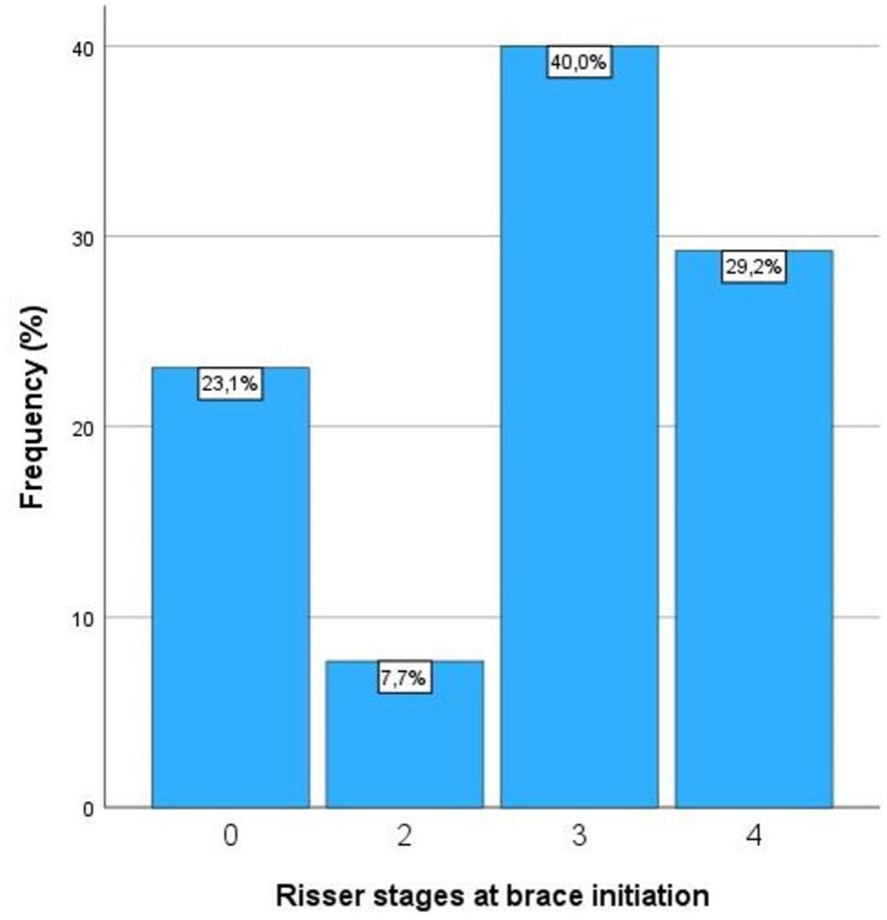
Distribution of Risser stages at brace initiation

There were 55 female (79.7%) and 14 male (20.3%) patients. The mean age at first presentation in our hospital was 13.7 ± 1.6 years (boys: 14.8 ± 1.4, girls: 13.5 ± 1.5 years, *p* = 0.005). The age reported by parents or patients when scoliosis first became apparent was 13.4 ± 1.7 years (boys: 14.3 ± 1.8 years, girls: 13.1 ± 1.6 years, *p* = 0.027). Age at brace initiation was 14.1 ± 1.5 years (boys: 15.1 ± 1.5 years, girls: 13.9 ± 1.4 years, *p* = 0.008). Age at menarche of included girls was 12.8 ± 1.2 years, and the period between this age and brace initiation was 1.1 ± 1.7 years (Table 1). The distribution of Risser stages are shown in Table 1 and Fig. 2. Period between first presentation and bracing start was 0.40 ± 0.65 years.

**Table 1:** Patient and curve characteristics of all included patients

	Male patients	Female patients	Total	
Included patient number	14 (20.3%)	55 (79.7%)	69 (100%)	
Patient characteristics				
Age at first presentation (years)	14.8 ± 1.4	13.5 ± 1.5	13.7 ± 1.6	*p* = 0.005
Age at first curve notation (years)	14.3 ± 1.8	13.1 ± 1.6	13.4 ± 1.7	*p* = 0.027
Age at brace initiation (years)	15.1 ± 1.5	13.9 ± 1.4	14.1 ± 1.5	*p* = 0.008
Age at menarche (years)	-	12.8 ± 1.2	-	–
Period menarche–brace initiation (years)	-	1.1 ± 1.7	-	–
Period menarche–brace termination (years)		3.7 ± 1.2		
Age at brace termination (years)	17.4 ± 1.3	16.6 ± 1.2	16.7 ± 1.3	*p* = 0.033
Period of bracing (years)	2.3 ± 1.3	2.7 ± 1.5	2.6 ± 1.5	*p* = 0.413
Risser stages				
0	4 (6.2%)	11 (16.9%)	15 (23.1%)	
2	2 (3.1%)	3 (4.6%)	5 (7.7%)	
3	6 (9.2%)	20 (30.8%)	26 (40.0%)	
4	2 (3.1%)	17 (26.1%)	19 (29.2%)	
Curve characteristics				
Cobb angle at initial presentation (°)	29.0 ± 9.8			
Cobb angle at brace initiation (°)	30.0 ± 9.2			
Cobb angle in best padded brace (°)	20.0 ± 11.8			
Cobb angle reduction in brace (%)	36.4 ± 31.8			
Cobb angle at brace termination (°)	29.9 ± 11.9			
Δ Cobb brace initiation–termination (°)	− 0.1 ± 7.8			
Curve pattern (n) thoracic/thoracolumbar/lumbar/combined	20/11/17/21			
Curve direction (n)^+^	20/1/19/1/13/4/4/7			
Nash & Moe 1/2/3 (n)	25/35/9			
Real brace wear 16–23 h/8–16 h/ < 8 h/brace refused (n)	21/25/22/1			

+ *thoracic right, lumbar left/thoracic left, lumbar right/thoracic right/thoracic left/lumbar left/lumbar right/thoracolumbar right/thoracolumbar left*

### Curve characteristics before bracing

In accordance with the Ponseti curve patterns, the distribution of curves across both genders were as follows: combined 30.4%, thoracic 29%, lumbar 24.6%, and thoracolumbar 15.9%. The ‘thoracic right/lumbar left’ curve was the most frequent curve direction (29%), followed by the ‘thoracic right’ with 27.5%, ‘lumbar left’ with 18.8%, and ‘thoracolumbar left’ with 10.1%.

**Fig. 3:**
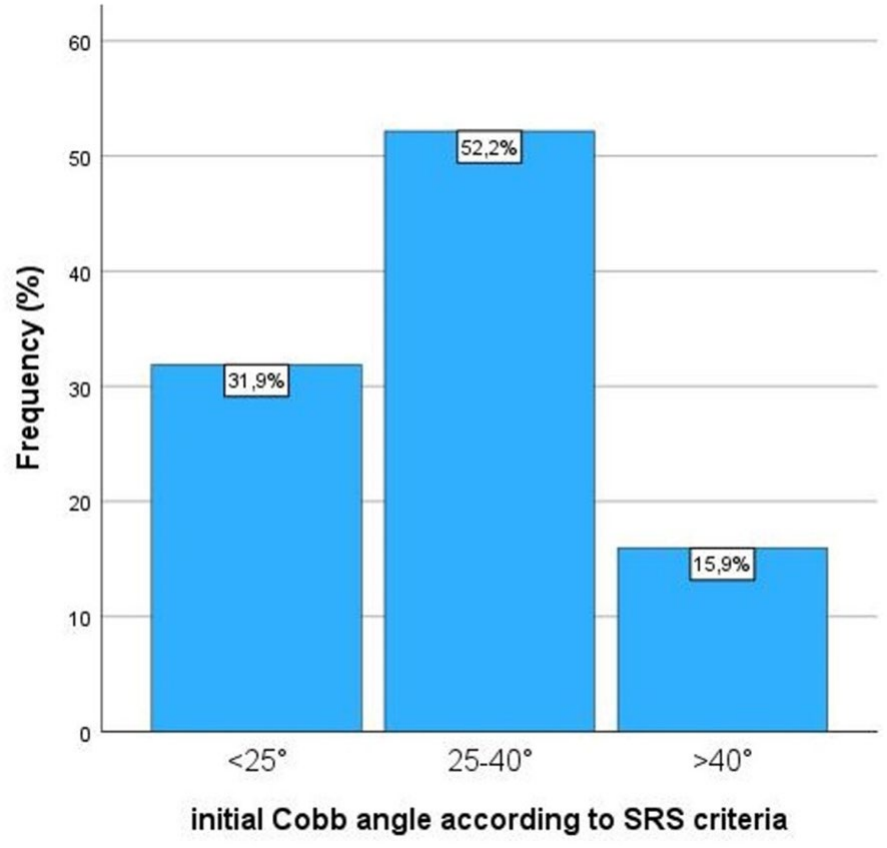
Frequency of initial Cobb angles according to SRS criteria

The Cobb angle at the initial presentation of patients was 29.0° ± 9.8° and at bracing start was 30.0° ± 9.2°. In 22 patients (31.9%), the Cobb angle was < 25°, in 36 patients (52.2%) between 25° and 40°, and in 11 patients (15.9%) > 40° (Fig. 3). Curve rotation according to Nash and Moe was Grade 2 in 35 patients (50.7%), Grade 1 in 25 (36.2%), and Grade 3 in nine patients (13.0%) (Table 1).

### Patients and curve characteristics during bracing

**Fig. 4:**
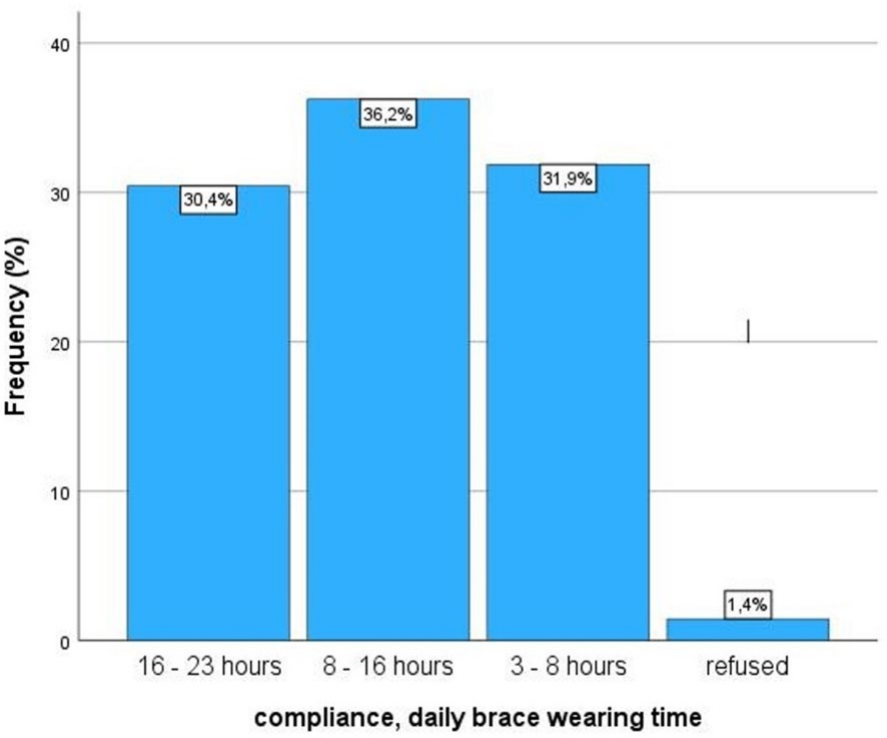
Real brace wear per day

**Fig. 5:**
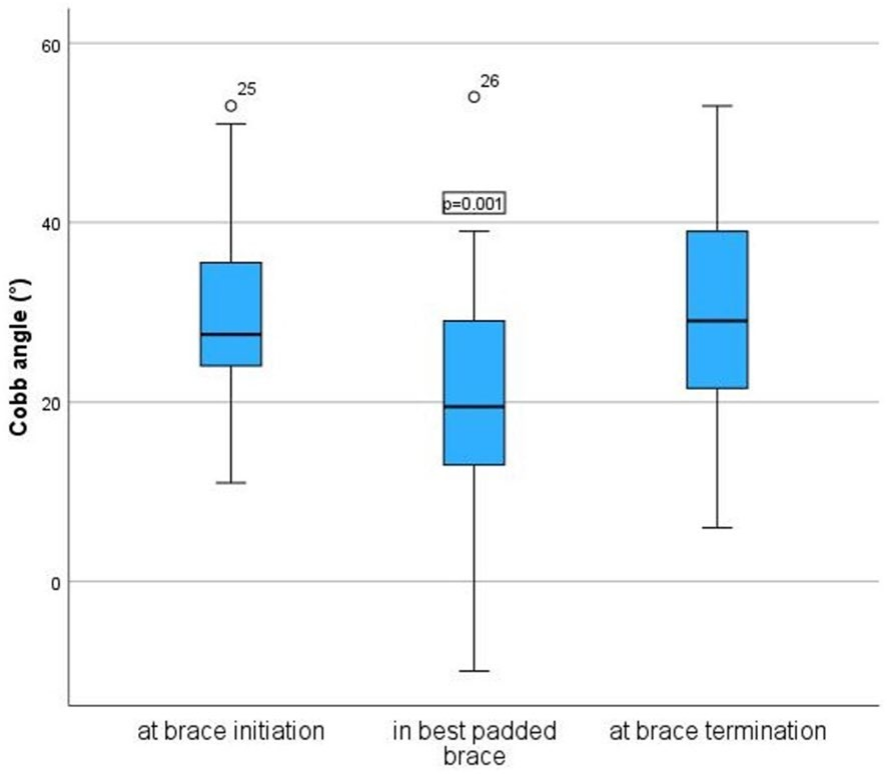
Cobb angle before, during and after bracing

The period of bracing was 2.6 ± 1.5 years and age at brace termination was 16.7 ± 1.3 years (girls: 16.6 ± 1.2 years, boys: 17.4 ± 1.3 years, *p* = 0.033). Bracing was terminated 3.7 ± 1.2 years after menarche. The RBW did not differ between groups (Fig. 4). The Cobb angle in the first brace after padding was significantly reduced to the initial Cobb angle (20.0° ± 11.8° vs 30.0° ± 9.2°, *p* < 0.001). Percentual in-brace-correction was 36.4% ± 31.8%. Cobb angle after brace termination was 29.9° ± 11.9°, which means there was no significant change to the Cobb angle before brace therapy (*p* = 0.903) (Fig. 5, Table 1).

### Outcome and determinants according to subgroups

#### All included patients (Supplement Tables 1–3)

**Fig. 6:**
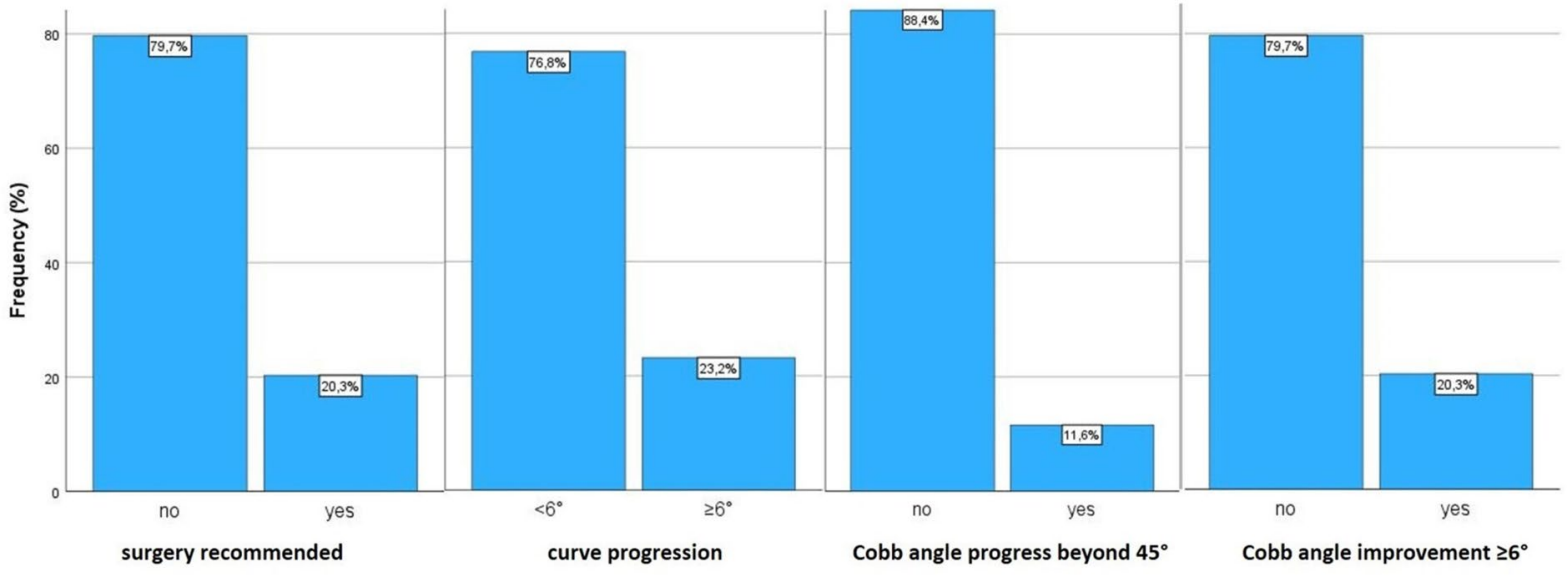
Outcome after bracing (all included patients)

After brace weaning related to all 69 included patients, surgery was recommended in 14 cases (20.3%), a curve progression of ≥ 6° was observed in 16 cases (23.2%), a Cobb angle beyond 45° was found in eight cases (11.6%), and a Cobb angle improvement of ≥ 6° was recorded in 14 patients (20.3%) (Fig. 6).

Patients with later indication for surgery had a significantly higher Cobb angle at initial presentation (38.1° ± 9.7° vs 26.6° ± 8.4°, *p* < 0.001) and at the beginning of bracing (38.8° ± 9.8° vs 27.8° ± 7.6°, *p* < 0.001). Furthermore, they had lower Risser stages at the start of bracing (*p* = 0.010) and higher degrees of rotation according to Nash and Moe (*p* = 0.030).

**Fig. 7:**
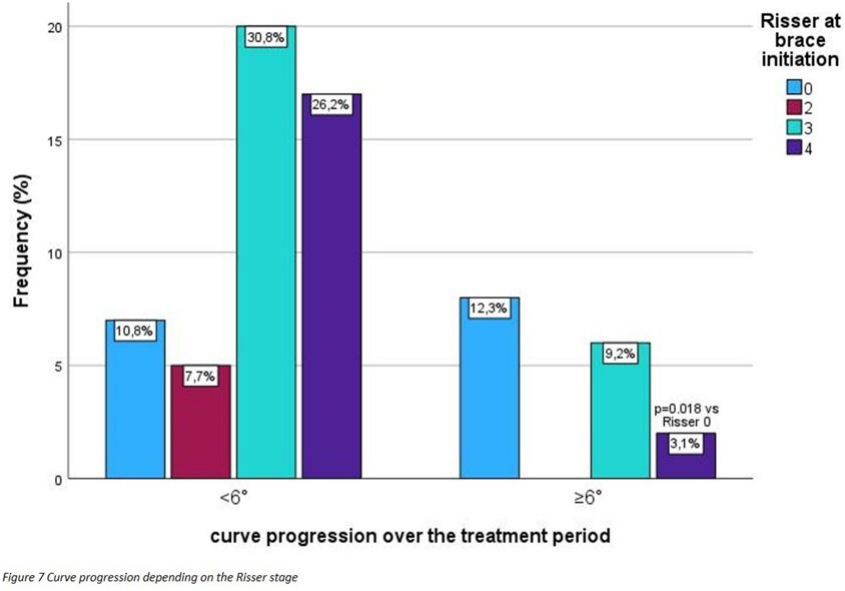
Curve progression depending on the Risser stage

Patients with Cobb angle progression of ≥ 6° at the end of bracing therapy were younger when the curve was initially noticed (12.4 ± 1.5 vs 13.7 ± 1.7 years, *p* = 0.011) and older when menarche occurred (13.4 ± 1.1 vs 12.6 ± 1.2 years, *p* = 0.037). The Risser sign was also shifted to smaller degrees (*p* = 0.015). There was a significantly diminished probability of curve progress ≥ 6° in the Risser 4 stage compared to Risser 0 (*p* = 0.018; Fig. 7). In the Risser 3 stage, there was no significantly reduced probability of curve progression compared to Risser 0. Nevertheless, 12.3% of all included patients still experienced curve progression ≥ 6° despite Risser 3 or 4 at brace initiation. This corresponds to 21.6% of all Risser 3 and 4 patients.

Cobb angle progression beyond 45° at the end of brace therapy was noted with higher initial Cobb angle values (45.0° ± 6.2° vs 28.0° ± 7.5°, *p* < 0.001) and higher initial Nash and Moe grades (*p* = 0.002).

Cobb angle reduction in brace (%) was the only significant different predictive variable comparing patients with Cobb angle improvement ≥ 6° vs < 6° (54.0% ± 31.2% vs 31.9% ± 30.7%; *p* = 0.019; Supplement Table 2).

RBW did not have a significant effect on the outcome parameters.

Detailed results of additionally conducted univariate binary logistic regression for significant different predictive variables are shown in Supplement Table 3.

#### Subgroup 1 (Supplement Tables 4 and 5)

Only nine (13.0%) of the included patients met the SRS research criteria. In three of those nine patients (33.3%), surgery was finally recommended, and curve progression was ≥ 6° in three patients (33.3%). There were no patients with curve progression beyond 45°. Cobb angle improvement ≥ 6° was observed in one patient (11.1%).

In the Mann–Whitney *U* test, the Cobb angle at brace initiation and initial presentation was significantly higher (36.7° ± 3.1° vs 28.8° ± 3.9°, *p* = 0.038 and *p* = 0.020, respectively) in patients for whom surgery later had to be recommended.

Patients with Cobb angle progression ≥ 6° at brace termination showed a significant difference of Nash and Moe grade compared with those with ≤ 5° progression (*p* = 0.048 in Fisher’s exact test).

#### Subgroup 2 (Supplement Table 6)

Twenty-two patients of the study population had initial Cobb degrees < 25° at brace initiation. In two patients (9.1%), surgery was finally recommended, curve progression was ≥ 6° in five patients (22.7%), and there was a curve improvement ≥ 6° in five patients (22.7%). There were no patients with curve progression beyond 45°.

The age of patients at brace initiation was significantly younger (11.7 ± 0.6 vs 14.3 ± 1.2 years; *p* = 0.017) in patients for whom surgery was recommended within the course.

There were no significantly different predictor variables in patients with final Cobb angle progress or improvement ≥ 6°.

#### Subgroup 3 (Supplement Table 7)

Eleven of our patients had Cobb angles > 40° at brace initiation. In seven patients (63.6%), surgery was finally recommended, and curve progression was ≥ 6° in four patients (36.4%). Of the six patients in this group in whom the Cobb angle was still in the range of 41°–44° at the start of bracing, three showed a Cobb angle progression beyond 45° at brace termination. Two patients (18.2%) had a Cobb angle improvement ≥ 6° over bracing time.

There were no statistical differences in the predictive parameters in patients for whom surgery later had to be recommended.

Patients with Cobb angle progression ≥ 6° at brace termination were significantly older at menarche (13.7 ± 1.0 vs 11.2 ± 1.1 years, *p* = 0.036) and showed a more frequent curve pattern favouring the thoracic and thoracolumbar curves (*p* = 0.036).

#### Subgroup outcome comparison (Table 2)

**Table 2:** Number of patients with the corresponding outcomes after brace weaning

Group/subgroup	Cobb angle progression < 6°	Cobb angle progression ≥ 6°	*p*	Surgery not recommended	Surgery recommended	*p*	Cobb progression not beyond 45°	Cobb progression beyond 45°	*p*	No Cobb angle improvement	Cobb angle improvement ≥ 6°	*p*
All patients	53 (76.8%)	16 (23.2%)		55 (79.7%)	14 (20.3%)		61 (88.4%)	8 (11.6%)		55 (79.7%)	14 (20.3%)	
SRS criteria fulfilling	6 (66.6%)	3 (33.3%)	*0.680 **0.660	6 (66.6%)	3 (33.3%)	*0.400 **0.131	9	0	*0.586	8 (88.9%)	1 (11.1%)	*1.00 **0.642
Initial Cobb degree < 25°	17 (77.3%)	5 (22.7%)	*1.00 ***0.438	20 (90.9%)	2 (9.1%)	*0.340 *** **0.002**	22	0	*0.191	17 (77.3%)	5 (22.7%)	*0.772 ***1.00
Initial Cobb degree > 40° (41–44°)	7 (63.6%)	4 (36.4%)	*0.454 ****1.00	4 (36.4%)	7 (63.6%)	*0.006 ****0.370	3 (50%)	3 (50%)	*0.038 ** **0.006** **** **0.044**	9 (81.8%)	2 (18.2%)	*1.00 ****1.00
Initial Cobb degree > 40° (≤ 45°)							–	–				

Fisher’s exact test: *to group of all patients

^**^ to subgroup Cobb degree < *25°*

^***^to subgroup initial Cobb degrees > *40°*

^****^ *to subgroup SRS criteria fulfilling; significant values in bold*

In all subgroups, RBW did not have a significant effect on the outcome parameters.

The comparison of the individual subgroups with the entire group of all patients and the comparison among the subgroups did not reveal any significant differences in the behaviour of curve progression < 6°/ ≥ 6° and curve improvement. Surgery was recommended more frequently to patients in the subgroup with an initial Cobb angle > 40° compared to all patients (*p* = 0.006) and compared to the subgroup with initial Cobb degree < 25° (*p* = 0.002).

Compared to all other subgroups and to the all-patients group, Cobb degree progression beyond 45° was significantly more frequent in the subgroup with initial Cobb angles > 40° (41°–44°; *p* = 0.006–*p* = 0.044).

## Discussion

Brace therapy is an established pillar during the conservative treatment of AIS, which is intended to prevent a poor outcome in the sense of severe curve progression and the need for surgery. Regarding the outcome of all patients treated with brace in this study, surgery was ultimately recommended in 20% of patients, curve progression was ≥ 6° in 23%, and the Cobb angle at brace termination was beyond 45° in 12%. These data are in line with recently published systematic reviews and studies [20–23]. In contrast to these undesirable outcome processes, we also observed a Cobb angle improvement ≥ 6° in 20% of cases.

More recent studies aim to adhere to the SRS research criteria when including their patients. We were unable to do this because the required criteria only applied to 13% of our treated patients. The same fact was observed in a recent study of three tertiary referral centres in the UK, where only 10% of patients met these criteria [24]. This might be due to different reasons. First, spinal curves with accompanying external body asymmetries in otherwise generally healthy adolescents provoke fears for parents and patients. Any therapy to prevent progress or surgery is welcome. Therefore, in case of doubt, the brace is prescribed to address all possible treatment options. Second, it can be assumed that due to organisational structures, patients arrive later in university hospitals and tertiary referral centres. The reasons for this may be that the disease is often initially treated by a specialist in private practice. Getting appointments in private practice takes time. Furthermore, there is another waiting time from the referral to the first appointment at the tertiary referral centre. Third, socioeconomic status seems to play a role in the severity of AIS at first presentation [25], although we did not specifically examine this in this study.

On the other hand, the following findings demonstrate that brace therapy can also be effective outside of the SRS criteria: First, as other authors also describe [26, 27], 21.6% of all our Risser 3 and 4 patients showed still curve progression of ≥ 6°. Second, in our study, we also included patients with a Cobb angle < 25° who received a brace. Like our results, Zapata et al. reported in patients braced at < 25° that 23% of curves improved at ≥ 6°, 25% progressed at ≥ 6° but did not require surgery, and 5% progressed to surgery. Third, we also found curve improvement in the group braced with initial Cobb > 40°. Heegard et al. [26] described in their study that progression risk in patients with curves exceeding 40° treated with night-time bracing is similar to smaller curves.

Taking these aspects into account, there is good reason to also research braced AIS patients who do not meet the SRS and SOSORT criteria and brace them in practice. However, it should be emphasized again in this context that the SRS criteria were primarily developed to compare the results of different braces [16]. They are not criteria for an indication for braces.

Moderate evidence suggests that brace wear time is associated with failure and success [28]. The review from Lee et al. [21] noted that in most publications, all patients with prescribed braces were included regardless of their compliance, so the impact of this important variable was not considered. Patient numbers in our three main groups of RBW were equally distributed. There was no significant difference in outcome in these groups. It must be noted that our study group may be too small to detect differences in a multifactorial influenced outcome. However, other authors with larger patient populations were also unable to find any influence of real brace wear [13] or did not evaluate it [29]. It can be assumed that all our examinations only provide correct results if the braces are actually worn. In our opinion, this is one of the most important parameters, as, otherwise, a mixture of treatment results with and without brace treatment will be obtained, which might best reflect reality. In a recently published systematic review, brace compliance has been shown to range from 7.0 to 18.8 h daily, and the proportion of compliant subjects in each study varies from 16.0% to 78.6% [30]. However, the aspect of the non-significant impact of brace-wearing time raises the question of whether brace therapy is effective at all.

Furthermore, in a recently published model of Dohan et al. called ‘BrAIST-Calc: Prediction of Individualized Benefit From Bracing for Adolescent Idiopathic Scoliosis’, duration of brace-wearing time per day was an included variable, which clearly underlines the relevance of this parameter. Further included parameters were age, sex, body mass index, Risser stage, and Cobb angle. The authors computed, among other things, the probability of prognosis in the dependency of brace-wearing time, including patients without brace and only observation. The authors did not recommend its use beyond their inclusion criteria, which essentially correspond to the SRS criteria and encourage additional investigation into the performance and utility of this model [31]. In conclusion, the discussion regarding under which conditions brace therapy is most effective is not over, and it can be assumed that brace wear time must be closely coordinated with other variables.

The same applies to the discussion of the predictor variable ‘in-brace correction’. Sato et al. computed that an in-brace major Cobb angle of < 25° in patients with immature skeletal status and < 30° in patients with mature skeletal structure should be aimed for achieving a clinically relevant success of brace treatment [32]. A systematic review by van den Bogaart et al. also found strong evidence that the lack of initial in-brace correction is associated with treatment failure. In patients of our study, the Cobb angle in best-padded braces was significantly higher in the group in which we later had to recommend surgery and in the group in which the Cobb angle ultimately developed to > 45°. There were no differences in this parameter when considering only the progression ≥ 6° vs < 6°. However, percentual in-brace correction itself was only a significant variable regarding curve improvement ≥ 6°. It must be noted that in-brace correction depends on the quality of the brace and the skills of the CPO but also on the flexibility of the curves [33], and this, in turn, probably depends on age, skeletal maturity, and other factors such as genetics.

The most likely reason for data inconsistency between studies are the multiple influence variables for the outcome of AIS. In our study, we demonstrated the influence of the following variables on the defined endpoints: Cobb angle at initial presentation and brace initiation, Risser sign, Nash and Moe degree, age at first curve notation and at brace initiation, in-brace correction, age at menarche, and curve pattern. Additional described influencing variables, which were not considered in our study, are BMI, sport and leisure engagement, genetic factors, bone mineral-density [34–39] and other radiographic parameters such as torsion, sagittal parameters, or intervertebral rotation. Improved treatment outcomes have also been reported when the braces were designed using CAD algorithms [40]. Last but not least, supplementary physiotherapy programs represent another pillar of treatment [41] and should be evaluated in detail. Due to this large number of influencing variables, in our opinion, only methods of big data management could provide more clarity as to which individual parameter constellation and with which dose a brace therapy will be successful. Further large multicentre prospective studies, possibly supported by machine learning (ML) or artificial intelligence (AI), could finally provide more valid statements and protect young people from unnecessary, unpopular brace therapies. The inclusion of previously less considered variables, such as genetic aspects, would then also be possible [42, 43]. Furthermore, research groups with smaller patient numbers should be encouraged to publish their treatment results so that their data can be incorporated into these systems. For Instance, Ohyama et al. presented an ML model showing that the most significant factors predicting curve progression varied for the different anatomical regions. For the advancement of proximal thoracic curves, the most important factor for prediction was the initial Cobb angle, whereas the presence of menarche was most relevant for main thoracic curves and the Risser grade for the progression of thoracolumbar/lumbar curves [44].

As discussed previously, the limitation of our study is the small number of cases for conducting multivariate regression models in a disease process influenced by many variables of which we could not capture all. On the other hand, precise data analysis over the course of brace therapy is one of our strengths and offers the possibility to compare our results to other recently published studies or include them in meta-analyses.

## Conclusion

While there was no significant influence of the RBW in all groups, the in-brace correction had a significant effect on the endpoint ‘curve improvement’ in the group including all patients. This confirms the effectiveness of brace therapy under certain conditions, although their complexity is not yet entirely clear. Depending on the nature of the patient groups and subgroups included in the respective statistics, we were able to prove the Cobb angle at initial presentation and at brace initiation, the Risser sign, the Nash and Moe degree, the age at first curve notation and at brace initiation, the in-brace correction, the age at menarche, and the curve pattern as predictive variables for outcome in our patients.

Big data management and AI for the purpose of evaluating the required larger amounts of data, including untreated patients without brace therapy, may be an approach to further investigate the multifactorial influenced aetiology and course of AIS in the future and for the development of prediction models. Until then, brace therapy for patients with AIS is definitely justified even outside the previously established criteria for a high risk of progression (above 10 years of age, Risser 0–2, curves 25°–40°) and may even lead to curve improvements. Furthermore, research in bracing outside these criteria should not be overlooked. Publishing research results with small patient numbers for data pooling should also be encouraged.

## Supplementary Information

Supplementary file 1

Supplementary file 2

Supplementary file 3

Supplementary file 4

Supplementary file 5

Supplementary file 6

Supplementary file 7

## Data Availability

Most of the data from the present study are shown in the supplement. Individual anonymised data for specific questions can be requested from the corresponding author.

## Abbreviations

AI: Artificial intelligence
AIS: Adolescent idiopathic scoliosis
CI: Confidence interval
CPO: Certified prosthetists orthoptist
DRG: Diagnosis-related groups
IBC: In-brace correction
ML: Machine learning
RBW: Real brace wear
SOSORT: International Society on Scoliosis Orthopaedic and Rehabilitation Treatment
SRS: Scoliosis Research Society
