# Supplementary material for: Results of bracing adolescent idiopathic scoliosis in the context of clinical practice and the Scoliosis Research Society’s criteria: 5-year observational study from a German orthopaedic university hospital
Source: Eur J Med Res. 2024 Oct 29;29:521. doi: 10.1186/s40001-024-02112-y (PMC11520584; doi:10.1186/s40001-024-02112-y)
Supplement: Supplementary file 1 [file 40001_2024_2112_MOESM1_ESM.docx]

Supplement, Table 1 Outcome and variables over all included 69 patients

| all included 69 patients | | | | | | | | | |
| --- | --- | --- | --- | --- | --- | --- | --- | --- | --- |
|  | surgery not recommended (n=55; ≙79.7%) | surgery recommended (n=14; ≙20.3%) | p | Cobb angle  progression ≤5°  (n=53; ≙76.8%) | Cobb angle progression ≥6° (n=16; ≙23.2%) | p | Cobb progression  not beyond 45°  (n=61; ≙88.4%) | Cobb progression beyond 45°  (n=8; ≙11.6%) | p |
| age at first presentation (years) | 13.8±1.6 | 13.6±1.2 | 0.641 | 13.9±1.6 | 13.3±1.4 | 0.224 | 13.7±1.6 | 13.8±0.9 | 0.838 |
| age at first curve notation (years) | 13.5±1.8 | 12.9±1.2 | 0.262 | 13.7±1.7 | 12.4±1.5 | **0.011** | 13.4±1.8 | 13.3±1.1 | 0.871 |
| age at menarche (years) | 12.6±1.0 | 13.3±1.7 | 0.266 | 12.6±1.2 | 13.4±1.1 | **0.037** | 12.8±1.1 | 12.5±1.6 | 0.517 |
| age at brace initiation (years) | 14.2±1.6 | 13.7±1.1 | 0.271 | 14.3±1.5 | 13.5±1.5 | 0.143** | 14.2±1.6 | 14.0±0.7 | 0.785 |
| age at brace termination (years) | 16.9±1.3 | 16.2±1.3 | 0.090 | 16.7±1.2 | 16.8±1.6 | 0.870 | 16.8±1.2 | 15.9±1.4 | 0.057 |
|  |  |  |  |  |  |  |  |  |  |
| Cobb angle at initial presentation (°) | 26.6±8.4 | 38.1±9.7 | **<0.001** | 28.5±10.2 | 30.8±8.5 | 0.422 | 27.0±8.3 | 43.9±7.2 | **<0.001** |
| Cobb angle at brace initiation (°) | 27.8±7.6 | 38.8±9.8 | **<0.001** | 29.5±9.3 | 31.6±8.8 | 0.358** | 28.0±7.5 | 45.0±6.2 | **<0.001** |
| Cobb angle in best padded brace (°) | 17.8±9.9 | 28.8±14.7 | **<0.001** | 19.5±10.5 | 21.6±15.7 | 0.541 | 17.8±10.4 | 36.5±8.6 | **<0.001** |
| Cobb angle reduction in brace (%) | 38.5±30.0 | 28.4±38.2 | 0.294 | 36.0±28.5 | 37.8±41.9 | 0.840** | 38.9±32.3 | 17.8±21.7 | 0.090** |
| Cobb angle at brace termination (°) | 25.9±9.5 | 45.6±6.1 | **<0.001** | 26.1±10.4 | 42.4±7.3 | **<0.001** | 27.2±9.9 | 50.1±2.7 | **<0.001** |
| Δ Cobb brace initiation – termination (°) | -1.9±7.1 | 6.9±6.9 | **<0.001** | -3.1±5.7 | 10.7±3.8 | **<0.001** | -0.8±7.7 | 5.1±7.7 | **0.044** |
|  |  |  |  |  |  |  |  |  |  |
| period brace time initiation – termination (years) | 2.6±1.5 | 2.5±1.6 | 0.741 | 2.4±1.2 | 3.3±2.2 | 0.355** | 2.7±1.5 | 1.9±1.2 | 0.187 |
| period menarche – brace initiation (years) | 1.3±1.6 | 0.6±2.1 | 0.248 | 1.5±1.5 | 0±2.1 | **0.008** | 1.1±1.7 | 1.8±1.5 | 0.334 |
| period menarche – brace termination (years) | 3.9±1.1 | 3.0±1.6 | **0.037** | 3.8±1.1 | 3.3±1.6 | 0.321 | 3.7±1.2 | 3.6±1.9 | 0.879 |
|  |  |  |  |  |  |  |  |  |  |
| gender male/female (n) | 10/45 | 4/10 | 0.388* | 11/42 | 3/13 | 0.861* | 12/49 | 2/6 | 0.725* |
| curve pattern (n) thoracic/thoracolumbar/lumbar/combined | 16/9/14/16 | 4/2/3/5 | 0.966* | 14/8/14/17 | 6/3/3/4 | 0.772* | 17/9/16/19 | 3/2/1/2 | 0.724* |
| curve direction^+^(n) | 16/0/15/1/12/2/3/6 | 4/1/4/0/1/2/1/1 | 0.345* | 17/0/13/1/11/3/2/6 | 3/1/6/0/2/1/2/1 | 0.379* | 19/0/16/1/13/3/3/6 | 1/1/3/0/0/1/1/1 | 0.098* |
| Nash & Moe 1/2/3 (n) | 22/29/4 | 3/6/5 | **0.030**** | 19/28/6 | 6/7/3 | 0.826** | 25/31/5 | 0/4/4 | **0.002**** |
| Risser at brace initiation 0/2/3/4 (n) | 7/5/22/17 | 8/0/4/2 | **0.010**** | 7/5/20/17 | 8/0/6/2 | **0.015**** | 12/5/23/17 | 3/0/3/2 | 0.661** |
| Real brace wear  16-23h/8-16h/<8h/brace refused (n) | 18/18/18/1 | 3/7/4/0 | 0.862** | 15/18/19/1 | 6/7/3/0 | 0.209** | 18/21/21/1 | 3/4/1/0 | 0.611** |
| *t-test unless otherwise marked; +thoracic right, lumbar left/thoracic left, lumbar right/thoracic right/thoracic left/lumbar left/lumbar right/thoracolumbar right/thoracolumbar left; * Chi-Square Tests;**Mann-Whitney Test; significant values in bold.* | | | | | | | | | |
