# Supplementary material for: Results of bracing adolescent idiopathic scoliosis in the context of clinical practice and the Scoliosis Research Society’s criteria: 5-year observational study from a German orthopaedic university hospital
Source: Eur J Med Res. 2024 Oct 29;29:521. doi: 10.1186/s40001-024-02112-y (PMC11520584; doi:10.1186/s40001-024-02112-y)
Supplement: Supplementary file 2 [file 40001_2024_2112_MOESM2_ESM.docx]

Supplement, Table 2 Cobb angle improvement and variables over all included 69 patients

| all included 69 patients | | | |
| --- | --- | --- | --- |
|  | Cobb angle improvement ≥6°  (n=14; ≙20.3%) | no Cobb angle improvement ≥6°  (n=55; ≙79.7%) | p |
| age at first presentation (years) | 13.4±1.9 | 13.8±1.5 | 0.362 |
| age at first curve notation (years) | 13.3 ±1.9 | 13.4±1.7 | 0.792 |
| age at menarche (years) | 12.2±1.3 | 12.9±1.1 | 0.062 |
| age at brace initiation (years) | 13.9±1.9 | 14.2±1.4 | 0.395** |
| age at brace termination (years) | 16.4±1.0 | 16.8±1.3 | 0.261 |
|  |  |  |  |
| Cobb angle at initial presentation (°) | 28.4±10.4 | 29.1±9.8 | 0.814 |
| Cobb angle at brace initiation (°) | 29.1±9.6 | 30.1±9.1 | 0.530** |
| Cobb angle in best padded brace (°) | 14.6±10.9 | 21.4±11.8 | 0.055 |
| Cobb angle reduction in brace (%) | 54.0±31.2 | 31.9±30.7 | **0.019**** |
| Cobb angle at brace termination (°) | 18.6±10.3 | 32.8±10.6 | **<0.001** |
| Δ Cobb brace initiation – termination (°) | -10.6±3.9 | 2.6±6.2 | **<0.001** |
|  |  |  |  |
| period brace time initiation – termination (years) | 2.5±1.4 | 2.6±1.5 | 0.911** |
| period menarche – brace initiation (years) | 1.2±1.8 | 1.1±1.7 | 0.875 |
| period menarche – brace termination (years) | 3.9±1.2 | 3.7±1.2 | 0.518 |
|  |  |  |  |
| gender male/female (n) | 2/12 | 12/43 | 0.532* |
| curve pattern (n) thoracic/thoracolumbar/lumbar/combined | 2/4/4/4 | 18/7/13/17 | 0.362* |
| curve direction^+^(n) | 4/0/2/0/2/2/1/3 | 16/1/17/1/11/2/3/4 | 0.509* |
| Nash & Moe 1/2/3 (n) | 6/7/1 | 19/28/8 | 0.448 |
| Risser at brace initiation 0/2/3/4 (n) | 3/1/5/4 | 12/4/21/15 | 0.938 |
| Real brace wear  16-23h/8-16h/<8h/brace refused (n) | 7/2/5/0 | 14/23/17/1 | 0.364 |
| *t-test unless otherwise marked; +thoracic right, lumbar left/thoracic left, lumbar right/thoracic right/thoracic left/lumbar left/lumbar right/thoracolumbar right/thoracolumbar left; * Chi-Square Tests;**Mann-Whitney Test; significant values in bold* | | | |
