# Supplementary material for: Results of bracing adolescent idiopathic scoliosis in the context of clinical practice and the Scoliosis Research Society’s criteria: 5-year observational study from a German orthopaedic university hospital
Source: Eur J Med Res. 2024 Oct 29;29:521. doi: 10.1186/s40001-024-02112-y (PMC11520584; doi:10.1186/s40001-024-02112-y)
Supplement: Supplementary file 3 [file 40001_2024_2112_MOESM3_ESM.docx]

*Supplement, Table 3 Results of univariate logistic regression for significant different predictive parameters (all 69 included patients)*

|  | OR | p | 95% CI | Omnibus | Hosmer and Lemeshow | Classification table (%) | Cox and Snel R square | Nagelkerke R square |
| --- | --- | --- | --- | --- | --- | --- | --- | --- |
| **Surgery recommended** | | | | | | | | |
| Cobb angle at brace initiation | 1.16 | <0.001 | 1.07-1.26 | <0.001 | 0.627 | 84.1 | 0.213 | 0.335 |
| Risser 3 vs 0 | 0.16 | 0.014 | 0.04-0.69 | 0.008 | 1 | 80.0 | 0.167 | 0.258 |
| Risser 4 vs 0 | 0.10 | 0.012 | 0.02-0.61 |  |  |  |  |  |
| Nash and Moe 1 vs 3 | 9.17 | 0.015 | 1.54-54.60 | 0.033 | 1 | 81.2 | 0.094 | 0.148 |
| **Cobb angle progression ≥6°** | | | | | | | | |
| age at first curve notation | 0.63 | 0.016 | 0.43-0.92 | 0.010 | 0.730 | 76.1 | 0.094 | 0.141 |
| age at menarche | 1.97 | 0.044 | 1.02-3.81 | 0.029 | <0.001 | 76.4 | 0.083 | 0.127 |
| Risser 3 vs 0 |  | n.s. |  |  |  |  |  |  |
| Risser 4 vs 0 | 0.10 | 0.012 | 0.02-0.61 | 0.012 | 1 | 76.9 | 0.155 | 0.230 |
| **Cobb angle progression beyond 45°** | | | | | | | | |
| Cobb angle at brace initiation | 1.34 | 0.002 | 1.12-1.60 | <0.001 | 0.927 | 94.2 | 0.314 | 0.613 |
| **Cobb angle improvement ≥ 6°** | | | | | | | | |
| Cobb angle reduction in brace (%) | 1.02 | 0.028 | 1.00-1.04 | 0.024 | 0.117 | 77.9 | 0.072 | 0.113 |
