# Supplementary material for: Results of bracing adolescent idiopathic scoliosis in the context of clinical practice and the Scoliosis Research Society’s criteria: 5-year observational study from a German orthopaedic university hospital
Source: Eur J Med Res. 2024 Oct 29;29:521. doi: 10.1186/s40001-024-02112-y (PMC11520584; doi:10.1186/s40001-024-02112-y)
Supplement: Supplementary file 4 [file 40001_2024_2112_MOESM4_ESM.docx]

Supplement, Table 4 Detailed data of patients meeting the SRS criteria

| gender | age at first presenta-tion (years) | age at first curve notation (years) | age at menarche (years) | Curve  pattern | Cobb  angle at brace  initiation (°) | age at brace  initiation (years) | Nash and Moe | Cobb  angle in best pad­ded brace (°) | Cobb  angle re­duction in brace (%) | Risser at brace initiation | brace wearing time  1≙16-23h  2≙8-16h  3≙<8h | Cobb  angle at brace ter­mination (°) | age at brace ter­mination (years) | surgery recom-mended | curve progres-sion ≥6° |
| --- | --- | --- | --- | --- | --- | --- | --- | --- | --- | --- | --- | --- | --- | --- | --- |
| f | 10.2 | 10.1 | 14.8 | combined | 25 | 10.4 | 1 | 6 | 76.0 | 0 | 1 | 36 | 17.5 | no | yes |
| f | 14.4 | 14.4 | 15.3 | combined | 40 | 14.6 | 2 | 29 | 27.5 | 0 | 2 | 40 | 17.3 | yes | no |
| f | 14.3 | 14.3 | 13.5 | combined | 35 | 14.6 | 2 | 28 | 20.0 | 2 | 1 | 30 | 17.1 | no | no |
| f | 12.0 | 11.6 | 13.2 | thoraco­lumbar | 25 | 12.2 | 2 | 18 | 28.0 | 0 | 1 | 6 | 16.9 | no | no |
| f | 11.3 | 10.8 | 11.8 | lumbar | 30 | 11.6 | 2 | 26 | 13.3 | 0 | 2 | 33 | 15.3 | no | no |
| f | 13.2 | 13.2 | 13.2 | combined | 27 | 13.6 | 1 | 18 | 33.3 | 2 | 2 | 28 | 15.5 | no | no |
| f | 12.8 | 12.3 | 13.4 | thoracic | 36 | 13.1 | 1 | 17 | 52.8 | 0 | 2 | 42 | 14.6 | yes | yes |
| f | 14.0 | 13.9 | 13.6 | lumbar | 31 | 14.5 | 2 | 13 | 58.1 | 2 | 3 | 30 | 15.9 | no | no |
| m | 14,.9 | 12.0 |  | combined | 34 | 15.3 | 1 | 32 | 5.9 | 0 | 3 | 44 | 16.4 | yes | yes |
| **Mean±SD** | **13.0±1,6** | **12.5±1.6** | **13.6±1.1** |  | **31.4±5.2** | **13.3±1.6** |  | **20.8±8.5** | **35.0±22.9** |  |  | **32.1±11.3** | **16.3±1.0** |  |  |
