# Supplementary material for: Results of bracing adolescent idiopathic scoliosis in the context of clinical practice and the Scoliosis Research Society’s criteria: 5-year observational study from a German orthopaedic university hospital
Source: Eur J Med Res. 2024 Oct 29;29:521. doi: 10.1186/s40001-024-02112-y (PMC11520584; doi:10.1186/s40001-024-02112-y)
Supplement: Supplementary file 5 [file 40001_2024_2112_MOESM5_ESM.docx]

*Supplement, Table 5 Outcome of patients meeting SRS criteria*

|  | patients meeting the SRS-criteria (above 10 years of age, Risser 0–2, curves 25-40°) | | | | | |
| --- | --- | --- | --- | --- | --- | --- |
|  | Cobb angle progression ≤5°  (n=6; ≙66.6%) | Cobb angle progression ≥6°  (n=3; ≙33.3%) | p | surgery not recommended (n=6; ≙66.6%) | surgery recommended (n=3; ≙33.3%) | p |
|  |  |  |  |  |  |  |
| age at first presentation (years) | 13.2±1.3 | 12.6±2.4 | 0.905 | 12.5±1.6 | 14.1±1.1 | 0.185 |
| age at first curve notation (years) | 13.0±1.5 | 11.4±1.2 | 0.262 | 12.3±1.7 | 12.9±1.3 | 0.625 |
| age at menarche (years) | 13.4±1.1 | 14.1±1.0 | 0.643 | 13.3±1.0 | 14.4±1.4 | 0.275 |
| age at brace initiation (years) | 13.5±1.3 | 12.9±2.4 | 0.905 | 12.8±1.7 | 14.3±1.1 | 0.210 |
| age at brace termination (years) | 16.3±0.9 | 16.2±1.5 | 1.00 | 16.4±0.9 | 16.1±1.4 | 0.735 |
|  |  |  |  |  |  |  |
| Cobb angle at initial presentation (°) | 31.3±5.5 | 31.7±5.9 | 0.905 | 28.8±3.9 | 36.7±3.1 | **0.020** |
| Cobb angle at brace initiation (°) | 31.3±5.5 | 31.7±5.9 | 0.905 | 28.8±3.9 | 36.7±3.1 | **0.038** |
| Cobb angle in best padded brace (°) | 22.0±6.5 | 18.3±13.1 | 0.714 | 18.2±8.2 | 26.0±7.9 | 0.214 |
| Cobb angle reduction in brace (%) | 30.0±15.4 | 44.9.5±35.7 | 0.714 | 38.1±24.1 | 28.7±23.5 | 0.595 |
| Cobb angle at brace termination (°) | 22.8±11.5 | 40.7±4.2 | **0.048** | 27.2±10.7 | 42.0±2.0 | **0.028** |
| Δ Cobb angle brace initiation – termination (°) | -3.5±8.0 | 9.0±2.6 | **0.024** | -1.7±10.0 | 5.3±5.0 | 0.302 |
|  |  |  |  |  |  |  |
| period brace time initiation – termination (years) | 2.8±1.2 | 3.2±3.40 | 0.714 | 3.6±2.1 | 1.8±0.8 | 0.210 |
| period menarche – brace initiation (years) | 0.1±0.9 | -2.3±2.9 | 0.286 | -0.5±2.0 | -0.5±0.4 | 0.990 |
| period menarche – brace termination (years) | 2.9±0.8 | 2.0±1.1 | 0.429 | 3.1±0.6 | 1.6±0.5 | **0.029** |
|  |  |  |  |  |  |  |
| gender male/female (n) | 0/6 | 1/2 | 0.333 | 0/6 | 1/2 | 0.333 |
| curve pattern thoracic/thoracolumbar/lumbar/combined (n) | 0/1/2/3 | 1/0/0/2 | 0.762 | 0/1/2/3 | 1/0/0/2 | 0.762 |
| curve direction^+^ | 3/0/0/0/2/0/1/0 | 2/0/1/0/0/0/0/0 | 0.762 | 3/0/0/0/2/0/1/0 | 2/0/1/0/0/0/0/0 | 0.762 |
| Nash & Moe 1/2/3 (n) | 1/5/0 | 3/0/0 | **0.048** | 2/4/0 | 2/1/0 | 0.524 |
| Risser at brace initiation 0/2 (n) | 3/3 | 3/0 | 0.464 | 3/3 | 3/0 | 0.464 |
| Real brace wear 16-23h/8-16h/<8h (n) | 2/3/1 | 1/1/1 | 1.00 | 3/2/1 | 0/2/1 | 0.500 |
| *+thoracic right, lumbar left/thoracic left, lumbar right/thoracic right/thoracic left/lumbar left/lumbar right/thoracolumbar right/thoracolumbar left (n); Mann-Whitney U test for metric variables, Fisher’s Exact Test for nominal and categorical variables; significant values in bold*  *No patients with Cobb angle progress beyond 45°. Only one patient with Cobb angle improvement. Therefore no further statistics for these aspects.* | | | | | | |
