# Supplementary material for: Results of bracing adolescent idiopathic scoliosis in the context of clinical practice and the Scoliosis Research Society’s criteria: 5-year observational study from a German orthopaedic university hospital
Source: Eur J Med Res. 2024 Oct 29;29:521. doi: 10.1186/s40001-024-02112-y (PMC11520584; doi:10.1186/s40001-024-02112-y)
Supplement: Supplementary file 6 [file 40001_2024_2112_MOESM6_ESM.docx]

*Supplement, Table 6 Outcome of with initial cobb degrees <25°*

|  | patients with initial cobb degrees <25° | | | | | | | | |
| --- | --- | --- | --- | --- | --- | --- | --- | --- | --- |
|  | Cobb angle progression ≤5°  (n=17; ≙77.3%) | Cobb angle progression ≥6°  (n=5; ≙22.7%) | p | surgery not recommended (n=20; ≙90.9%) | surgery recommended (n=2; ≙9.1%) | p | Cobb angle improvement ≥6°(n=5; ≙22.7%) | no Cobb angle improvement ≥6° (n=17; ≙77.3%) | p |
|  |  |  |  |  |  |  |  |  |  |
| age at first presentation (years) | 13.7±1.6 | 12.5±1.7 | 0.218 | 13.7±1.6 | 11.4±0.5 | 0.104 | 13.9±1.7 | 13.3±1.7 | 0.493 |
| age at first curve notation (years) | 13.5±1.8 | 11.6±1.6 | 0.053 | 13.2±1.9 | 11.4±0.5 | 0.211 | 13.8±1.7 | 12.8±2.0 | 0.306 |
| age at menarche (years) | 12.8±0.9 | 13.0±1.1 | 0.785 | 12.7±0.8 | 14.6±0 | 0.118 | 13.0±0.4 | 12.8±1.0 | 0.412 |
| age at brace initiation (years) | 14.4±1.1 | 12.8±1.6 | 0.085 | 14.3±1.2 | 11.7±0.6 | **0.017** | 14.7±1.1 | 13.9±1.4 | 0.359 |
| age at brace termination (years) | 16.7±1.2 | 17.4±0.6 | 0.319 | 16.9±1.2 | 17.1±0.3 | 0.866 | 16.9±0.7 | 16.9±1.3 | 0.649 |
|  |  |  |  |  |  |  |  |  |  |
| Cobb angle at initial presentation (°) | 19.6±4.0 | 21.8±3.3 | 0.319 | 19.8±4.0 | 23.0±1.4 | 0.312 | 18.4±2.9 | 20.6±4.1 | 0.140 |
| Cobb angle at brace initiation (°) | 20.7±3.3 | 21.8±3.3 | 0.401 | 20.7±3.3 | 23.0±1.4 | 0.312 | 20.2±1.3 | 21.1±3.7 | 0.218 |
| Cobb angle in best padded brace (°) | 12.0±8.1 | 5.8±10.8 | 0.283 | 11.4±7.9 | 3.0±18.4 | 0.623 | 6.0±8.7 | 11.9±8.7 | 0.189 |
| Cobb angle reduction in brace (%) | 42.6±37.8 | 74.3±46.5 | 0.189 | 46.4±37.2 | 84.5±80.9 | 0.554 | 71.0±42.8 | 43.6±39.7 | 0.189 |
| Cobb angle at brace termination (°) | 17.1±6.4 | 34.8±2.9 | **<0.001** | 19.6±8.5 | 36.5±3.5 | **0.035** | 10.6±2.3 | 24.2±8.5 | **<0.001** |
| Δ Cobb angle brace initiation – termination (°) | -3.5±5.0 | 13.0±4.1 | **<0.001** | -1.1±7.7 | 13.5±2.1 | **0.035** | -9.6±2.3 | 3.1±7.4 | **<0.001** |
|  |  |  |  |  |  |  |  |  |  |
| period brace time initiation – termination (years) | 2.3±1.1 | 4.7±1.9 | **0.006** | 2.6±1.4 | 5.4±0.9 | **0.035** | 2.2±1.0 | 3.0±1.7 | 0.359 |
| period menarche – brace initiation (years) | 1.4±1.0 | -0.1±2.5 | 0.412 | 1.3±1.1 | -3.4±0 | 0.118 | 1.3±0.8 | 1.0±1.7 | 0.871 |
| period menarche – brace termination (years) | 3.5±0.7 | 4.5±1.4 | 0.202 | 3.8±1.0 | 2.7±0 | 0.235 | 3.7±0.4 | 3.8±1.1 | 0.956 |
|  |  |  |  |  |  |  |  |  |  |
| gender male/female (n) | 5/12 | 1/4 | 1.00 | 5/15 | 1/1 | 0.481 | 1/4 | 5/12 | 1.00 |
| curve pattern thoracic/thoracolumbar/lumbar/combined (n) | 5/2/5/5 | 1/1/2/1 | 1.00 | 6/3/6/5 | 0/0/1/1 | 1.00 | 0/2/1/2 | 6/1/6/4 | 0.122 |
| curve direction^+^ (n) | 5/0/4/1/5/0/0/2 | 1/0/1/0/1/1/1/0 | 0.413 | 5/0/5/1/6/0/1/2 | 1/0/0/0/0/1/0/0 | 0.264 | 2/0/0/0/1/0/0/2 | 4/0/5/1/5/1/1/0 | 0.178 |
| Nash & Moe 1/2/3 (n) | 11/6/0 | 2/2/1 | 0.226 | 12/8/0 | 1/0/1 | 0.091 | 4/1/0 | 9/7/1 | 0.696 |
| Risser at brace initiation 0/2/3/4 (n) | 2/2/6/4 | 3/0/2/0 | 0.203 | 3/2/8/4 | 2/0/0/0 | 0.205 | 1/1/2/0 | 4/1/6/4 | 0.690 |
| Real brace wear 16-23h/8-16h/<8h (n) | 2/6/9 | 2/2/1 | 0.272 | 4/7/9 | 0/1/1 | 1.00 | 1/1/3 | 3/7/7 | 0.809 |
| *+thoracic right, lumbar left/thoracic left, lumbar right/thoracic right/thoracic left/lumbar left/lumbar right/thoracolumbar right/thoracolumbar left; Mann-Whitney U test for metric variables, Fisher’s Exact Test for nominal and categorical variables; significant values in bold*  *No patients with Cobb angle progress beyond 45°. Therefore no statistics for this aspect.*  *Detailed data cause of larger amount not provided here. If of interest contact the corresponding author.* | | | | | | | | | |
