# Supplementary material for: Results of bracing adolescent idiopathic scoliosis in the context of clinical practice and the Scoliosis Research Society’s criteria: 5-year observational study from a German orthopaedic university hospital
Source: Eur J Med Res. 2024 Oct 29;29:521. doi: 10.1186/s40001-024-02112-y (PMC11520584; doi:10.1186/s40001-024-02112-y)
Supplement: Supplementary file 7 [file 40001_2024_2112_MOESM7_ESM.docx]

*Supplement, Table 7 Outcome of patients with Cobb angles >40° at brace initiation*

| patients with Cobb angle >40°at brace initiation (n=11) | | | | | | |
| --- | --- | --- | --- | --- | --- | --- |
|  | Cobb angle progression ≤5° (n=7; ≙63.6%) | Cobb angle progression ≥6° (n=4; ≙36.4%) | p | surgery not recommended (n=4; ≙36.4%) | surgery recommended (n=7; ≙63.6%) | p |
|  |  |  |  |  |  |  |
| age at first presentation (years) | 13.7±1.2 | 14.3±0.7 | 0.315 | 13.8±1.5 | 14.0±0.8 | 0.648 |
| age at first curve notation (years) | 13.6±1.2 | 13.2±1.4 | 1.00 | 13.7±1.5 | 13.4±1.1 | 0.788 |
| age at menarche (years) | 11.2±1.1 | 13.7±1.0 | **0.036** | 11.4±1.2 | 12.5±1.8 | 0.393 |
| age at brace initiation (years) | 14.0±1.2 | 14.4±0.4 | 0.230 | 14.1±1.6 | 14.1±0.7 | 0.527 |
| age at brace termination (years) | 16.2±0.9 | 16.2±1.8 | 0.527 | 16.3±1.3 | 16.2±1.3 | 0.788 |
|  |  |  |  |  |  |  |
| Cobb angle at initial presentation (°) | 47.1±4.9 | 40.8±4.2 | 0.109 | 44.0±4.1 | 45.3±6.4 | 0.648 |
| Cobb angle at brace initiation (°) | 47.1±4.9 | 43.0±1.8 | 0.315 | 44.0±4.1 | 46.6±4.7 | 0.315 |
| Cobb angle in best padded brace (°) | 34.4±4.4 | 35.3±12.8 | 0.648 | 31.8±3.9 | 36.4±9.3 | 0.315 |
| Cobb angle reduction in brace (%) | 26.9±6.9 | 18.4±28.0 | 0.927 | 27.6±9.2 | 21.6±20.4 | 0.527 |
| Cobb angle at brace termination (°) | 42.9±5.8 | 51.5±1.3 | **0.024** | 39.0±3.5 | 50.0±2.9 | **0.006** |
| Δ Cobb angle brace initiation – termination (°) | -4.3±3.4 | 8.5±1. 0 | **0.006** | -5.0±4.2 | 3.4±6.5 | 0.073 |
|  |  |  |  |  |  |  |
| period brace time initiation – termination (years) | 2.3±0.7 | 1.8±1.5 | 0.230 | 2.1±0.7 | 1.1±1.2 | 0.788 |
| period menarche - brace initiation (years) | 2.6±1.2 | 0.9±0.8 | 0.143 | 1.9±0.9 | 1.9±1.7 | 1.00 |
| period menarche – brace termination (years) | 4.7±1.4 | 3.0±1.7 | 0.230 | 4.2±1.6 | 4.0±1.9 | 1.00 |
|  |  |  |  |  |  |  |
| gender male/female (n) | 2/5 | 1/3 | 1.00 | 1/3 | 2/5 | 1.00 |
| curve pattern thoracic/thoracolumbar/lumbar/combined (n) | 1/0/2/4 | 2/2/0/0 | **0.036** | 0/0/1/3 | 3/2/1/1 | 0.218 |
| curve direction^+^ (n) | 4/1/1/1/0/0 | 0/2/0/0/1/1 | 0.109 | 3/0/0/0/1/0/0/0 | 1/0/3/0/0/1/1/1 | 0.218 |
| Nash & Moe 1/2/3 (n) | 0/4/3 | 0/2/2 | 1.00 | 0/3/1 | 0/3/4 | 0.545 |
| Risser at brace initiation 0/2/3/4 (n) | 1/0/4/2 | 2/0/1/1 | 0.727 | 0/0/3/1 | 3/0/2/2 | 0.455 |
| Real brace wear 16-23h/8-16h/<8h/brace refused (n) | 3/1/3/0 | 2/2/0/0 | 0.455 | 2/0/2/0 | 3/3/1/0 | 0.455 |
| +thoracic right, lumbar left/thoracic left, lumbar right/thoracic right/thoracic left/lumbar left/lumbar right/thoracolumbar right/thoracolumbar left; Mann-Whitney U test for metric variables, Fisher’s Exact Test for nominal and categorical variables, significant values in bold.  *The statistical evaluation of predictive parameters regarding Cobb angle beyond 45° and curve improvement was not carried out in this patient group due to lack of meaningfulness..* | | | | | | |
